# Mitigating Doxorubicin‐Induced Cardiotoxicity and Enhancing Anti‐Tumor Efficacy with a Metformin‐Integrated Self‐Assembled Nanomedicine

**DOI:** 10.1002/advs.202415227

**Published:** 2025-03-07

**Authors:** Jiaxin Huang, Jieru Yang, Yuanying Yang, Xiaofeng Lu, Juan Xu, Shan Lu, Hong Pan, Wenhu Zhou, Wenqun Li, Songwen Chen

**Affiliations:** ^1^ Department of Pharmacy Second Xiangya Hospital Central South University Changsha Hunan 410011 China; ^2^ Xiangya School of Pharmaceutical Sciences Central South University Changsha Hunan 410013 China; ^3^ Department of Cardiology Shanghai General Hospital Shanghai Jiao Tong University School of Medicine No.100, Haining Rd Shanghai 200080 China; ^4^ Department of Cardiology Shanghai General Hospital Jiuquan Hospital No. 22, West St Jiuquan Gansu 735000 China; ^5^ Hunan Key Laboratory of The Research and Development of Novel Pharmaceutical Preparations School of Pharmaceutical Science Changsha Medical University Changsha Hunan 410219 China

**Keywords:** cGAS‐STING, immunotherapy, mitochondrial dysfunction, nanoparticles, targeting, tumor therapy

## Abstract

Doxorubicin (Dox) is a potent chemotherapeutic agent commonly used in cancer treatment. However, cardiotoxicity severely limited its clinical application. To address this challenge, a novel self‐assembled nanomedicine platform, PMDDH, is developed for the co‐delivery of Dox and metformin, an antidiabetic drug with cardioprotective and anti‐tumor properties. PMDDH integrates metformin into a polyethyleneimine‐based bioactive excipient (PMet), with Dox intercalated into double‐stranded DNA and a hyaluronic acid (HA) coating to enhance tumor targeting. The PMDDH significantly improves the pharmacokinetics and tumor‐targeting capabilities of Dox, while metformin enhances the drug's anti‐tumor activity by downregulating programmed cell death ligand 1 (PD‐L1) and activating the AMP‐activated protein kinase (AMPK) signaling pathway. Additionally, the DNA component stimulates the cyclic GMP‐AMP synthase‐stimulator of interferon genes (cGAS‐STING) pathway, which synergizes with Dox‐induced immunogenic cell death (ICD) to promote a robust anti‐tumor immune response. PMDDH markedly reduces Dox‐induced cardiotoxicity by preserving mitochondrial function, reducing reactive oxygen species (ROS) production, and inducing protective autophagy in cardiomyocytes. These findings position PMDDH as a promising dual‐function nanomedicine that enhances the anti‐tumor efficacy of Dox while minimizing its systemic toxicity, offering a safer and more effective alternative for cancer therapy.

## Graphical abstract

A novel self‐assembled nanomedicine (PMDDH) has been developed for the co‐delivery of doxorubicin and metformin, aiming to enhance the anti‐tumor efficacy of doxorubicin while alleviating its cardiotoxicity through the activation of cGAS‐STING and AMPK signaling pathways, respectively. This innovative strategy addresses the critical demand for safer and more effective tumor therapies, providing a promising multi‐functional platform for clinical translation.

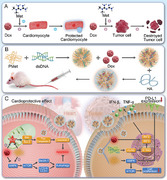

## 1 Introduction

Malignant tumors are a significant global health burden and one of the most formidable challenges in modern medicine.^[^ ^1^ ^]^ Chemotherapy remains a cornerstone of cancer treatment, employing cytotoxic drugs to eradicate tumor cells, and is recommended as a first‐line therapy in clinical guidelines worldwide.^[^ ^2^ ^]^ Doxorubicin (Dox), an anthracycline chemotherapeutic agent, is widely used due to its broad anti‐tumor spectrum and potent cytotoxic effects, effectively treating breast, ovarian, and various other cancers.^[^ ^3a,b^ ^]^ However, the dose‐dependent side effects severely limit its clinical application, particularly bone marrow suppression and cardiotoxicity.^[^ ^4^ ^]^ Dox‐induced cardiotoxicity (DIC) is especially problematic, often manifesting within the first year of treatment and significantly impacting patient prognosis and allowable dosage.^[^ ^5^ ^]^ Thus, enhancing the therapeutic efficacy of Dox while minimizing its cardiotoxicity is crucial for improving patient compliance and outcomes.

Multidrug delivery systems have emerged as a promising strategy to overcome the limitations of single‐agent chemotherapy by optimizing drug efficacy and minimizing side effects through rational drug combinations.^[^ ^6a,b^ ^]^ For example, combining Brentuximab Vedotin with Dox significantly prolongs progression‐free survival in lymphoma patients,^[^ ^7^ ^]^ and pretreatment of MDA‐MB‐231 cells with alkaloids such as Piperine enhances their sensitivity to Dox.^[^ ^8^ ^]^ Notably, Dox also induces immunogenic cell death (ICD), facilitating the release of danger‐associated molecular patterns (DAMPs) such as adenosine triphosphate (ATP), high‐mobility group protein B1 (HMGB1), and calreticulin (CRT) from dying cells, thereby enhancing antigen presentation and T cell activation.^[^ ^9a,b^ ^]^ This immunomodulatory effect has spurred research into combining Dox with immunotherapeutic strategies, including nanocarriers that co‐deliver Dox alongside immune modulators to reprogram the tumor microenvironment.

Nanoparticle drug delivery systems provide a robust platform to improve chemotherapeutic agents' pharmacokinetics, biodistribution, and safety profiles.^[^ ^10a,b^ ^]^ Encapsulating drugs in nanocarriers can enhance stability, prolong circulation time, and increase accumulation at tumor sites via the enhanced permeability and retention (EPR) effect.^[^ ^11^ ^]^ Recent innovations include integrating auxiliary drugs into carrier materials to create bioactive excipients that enhance therapeutic efficacy.^[^ ^12a,b^ ^]^ Despite these advancements, systemic toxicity remains a significant barrier to clinical translation. For instance, although the FDA has approved dexmedetomidine to reduce Dox‐induced cardiotoxicity, its side effects and potential tumor‐promoting risks limit its clinical application.^[^ ^13a,b^ ^]^ Consequently, there is an urgent need for safer and more effective multidrug delivery systems that can enhance the therapeutic index of Dox while mitigating its adverse effects.

Emerging evidence suggests that metformin, a first‐line antidiabetic drug, may mitigate DIC by activating AMP‐activated protein kinase (AMPK) signaling, regulating redox balance, and inhibiting mitochondrial dysfunction and apoptosis induced by excessive autophagy.^[^ ^14a,b^ ^]^ Clinical studies have demonstrated that metformin can reduce mitochondrial dysfunction in breast cancer patients treated with Dox.^[^ ^15^ ^]^ Moreover, metformin exhibits intrinsic anti‐tumor effects, including inhibition of tumor proliferation via AMPK activation and mammalian target of rapamycin (mTOR) pathway suppression.^[^ ^16^ ^]^ Notably, metformin also promotes the degradation of programmed cell death ligand 1 (PD‐L1) on tumor cells, enhancing anti‐tumor immunity.^[^ ^17^ ^]^ These findings highlight the potential of metformin as a bioactive agent to improve the therapeutic efficacy of Dox while reducing its cardiotoxicity.

**Scheme 1:**
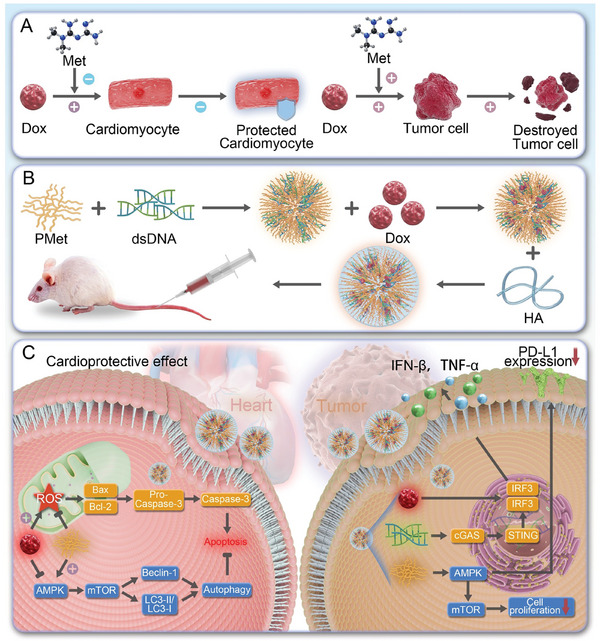
Design and mechanism of PMDDH. A) Schematic representation of metformin enhancing the cytotoxic effect of Dox on 4T1 cells and mitigating its cardiotoxicity on H9c2 cells. B) Synthesis of PMD, PMDD, and PMDDH. C) Illustration of the targeted delivery, enhanced therapeutic efficacy, and reduced cardiotoxicity of PMDDH in vivo.

Based on these insights, this study investigates the use of metformin as a bioactive excipient to enhance the therapeutic index of Dox. At the cellular level, we demonstrate that metformin not only augments the cytotoxicity of Dox against 4T1 breast cancer cells but also reduces its cardiotoxicity on H9c2 cardiomyocytes (**Scheme** **1**A), supporting the rationale for their combined use. Metformin was integrated into linear polyethyleneimine (PEI) to form the bioactive excipient PMet to achieve effective co‐delivery. The PMet self‐assembled with double‐stranded DNA (dsDNA), with Dox intercalated within the DNA structure, to construct PMDDH nanoparticles. Finally, hyaluronic acid (HA) was adsorbed onto the nanoparticle surface (Scheme 1B).

Our results demonstrate that PMDDH nanoparticles offer significant advantages over free Dox (Scheme 1C). The nanodrug delivery system not only improves the pharmacokinetics and tumor‐targeting capabilities of Dox but also retains the activity of metformin, enhancing Dox's anti‐tumor effects. Moreover, PMet downregulates PD‐L1 via AMPK activation, while the DNA component activates the cyclic GMP‐AMP synthase‐stimulator of interferon genes (cGAS‐STING) signaling pathway, complementing the ICD effect of Dox. Together, these mechanisms promote macrophage polarization, dendritic cell (DCs) activation, and CD8^+^ T‐cell infiltration, effectively transforming “cold tumors” into “hot tumors” and amplifying the anti‐tumor immune response. Significantly, PMDDH also mitigates DIC by reducing reactive oxygen species (ROS) production, preserving mitochondrial function, and inducing protective autophagy via the AMPK‐mTOR pathway, thereby offering substantial cardioprotection in vivo.

This study introduces a novel self‐assembled nanomedicine for the co‐delivery of Dox and metformin, providing a new strategy to enhance the therapeutic efficacy and reduce the toxicity of Dox in cancer therapy. Integrating bioactive excipients in the design and optimization of this nanocarrier system represents an innovative approach to addressing current challenges in cancer treatment. Our findings offer a compelling rationale for the further development and clinical translation of PMDDH as a safer and more effective alternative to conventional chemotherapeutic regimens.

## 2 Results and Discussion

### 2.1 Construction and Characterization of PMDDH

**Figure 1:**
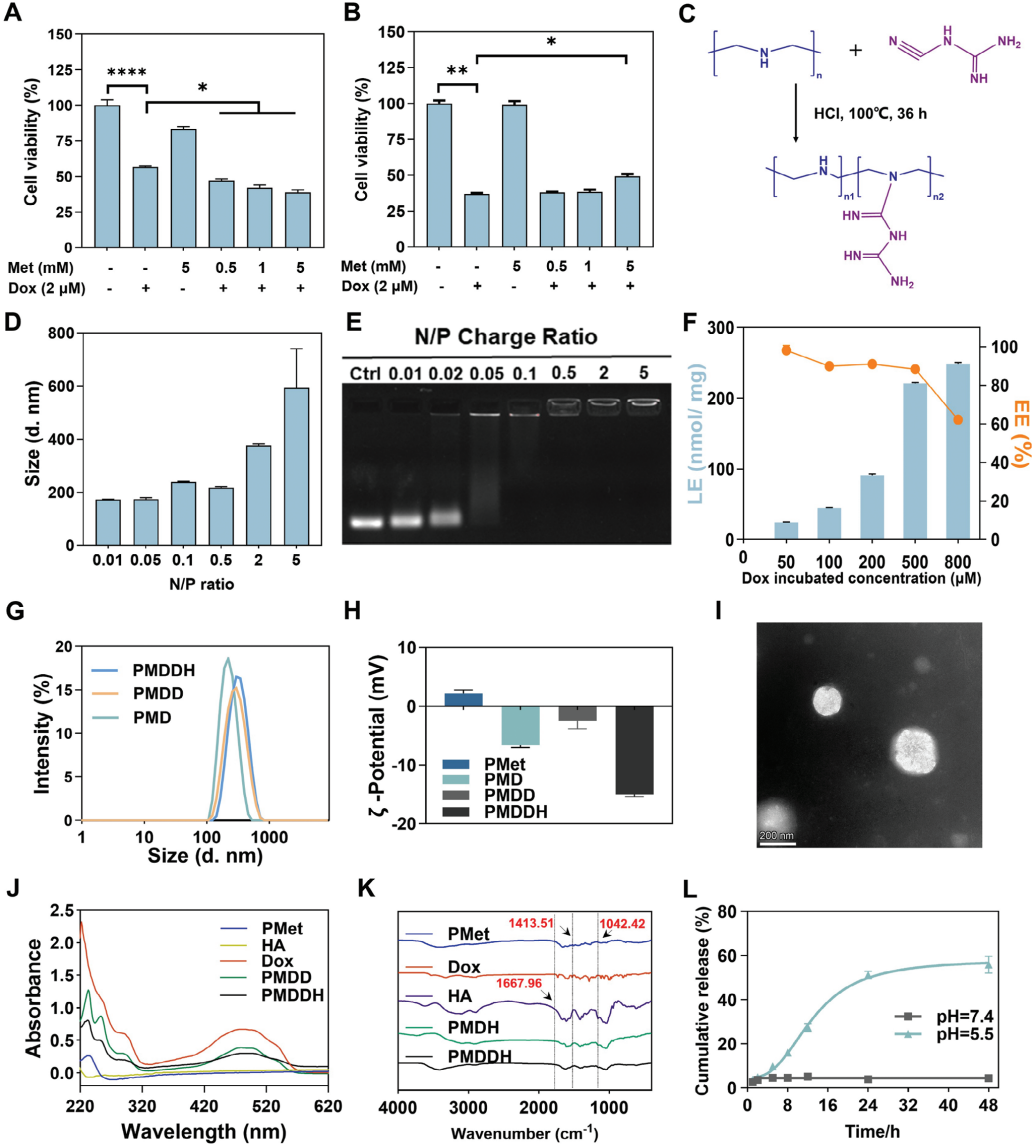
Cytotoxicity of A) MCF‐7 and B) H9c2 cells treated with Dox and metformin (*n* = 5). C) Schematic illustration of PMet synthesis. D) The particle size of PMD at varying N/P ratios. E) DNA loading efficiency of PMD assessed by agarose gel electrophoresis. F) Drug loading efficiency (LE%) and encapsulation efficiency (EE%) of PMDDH at different Dox concentrations (*n* = 3). G) Size distribution of PMD, PMDD, and PMDDH. H) ζ‐potential of PMet, PMD, PMDD, and PMDDH (*n* = 3). I) TEM micrograph of PMDDH (negative staining with 2% phosphotungstic acid). J) UV–vis spectra of PMet, HA, Dox, PMDD, and PMDDH. K) FTIR spectra of PMet, Dox, HA, PMDH, and PMDDH. L) Dox release profiles of PMDDH under different pH conditions (*n* = 3). Data are presented as mean ± SD (*p < 0.05, **p < 0.01, ***p < 0.001, ****p < 0.0001).

To explore the potential of metformin in enhancing the detoxification of Dox at the cellular level, we performed CCK‐8 assays. The results demonstrated that metformin significantly enhanced the cytotoxicity of Dox on MCF‐7 breast cancer cells (**Figure** **1**A) while concurrently mitigating its toxic effects on H9c2 cardiomyocytes (Figure 1B). Notably, the modulatory effects of metformin were dose‐dependent within a specific concentration range, and metformin alone did not significantly affect cell proliferation even at 5 mm. These findings provide a rationale for the co‐delivery of metformin and Dox despite the challenges posed by their distinct physicochemical properties. To address these challenges, we developed a ternary self‐assembly system using DNA as a bridging agent to facilitate the combined delivery of both drugs (Scheme 1B).

Figure 1C depicts polymetformin (PMet), a metformin polymer synthesized via the Pinner reaction. The chemical structure of PEI and PMet were confirmed using ^1^H NMR (400 MHz) (Figure S1A,B, Supporting Information). The peak observed at 4.7 ppm corresponds to water, whereas the peaks at 3.5 ppm were attributed to hydrogen atoms at position a (─CH_2_─) of PEI. In the spectrum of PMet, the peaks observed at 2.51 ppm correspond to DMSO, while the peaks at 2.88 and 4.06 ppm are attributed to the hydrogen atoms at positions a‐c (N─CH_2_). The peak at 6.21 ppm is assigned to hydrogen atoms at position g (─NH_2_), and the peaks at 7.08 and 8.03 ppm correspond to hydrogen atoms at positions d‐f (─NH). Subsequent MALDI‐TOF mass spectrometry analysis showed that the distribution center shifted to 2353 Da (Figure S1C, Supporting Information), consistent with previous literature reports, confirming the successful synthesis of PMet.^[^ ^18^ ^]^ The number‐average molecular weight of PMet was determined to be 1656 g mol^−1^ by gel permeation chromatography (Figure S2, Supporting Information), corresponding to ≈12 metformin monomer units per PMet molecule.

PMet, as a cationic polymer, formed nanocarriers (PMD) with negatively charged DNA through electrostatic interactions. By fixing the DNA concentration and varying the N/P ratio of PMet, it was observed that PMD maintained a particle size of ≈200 nm when the N/P ratio was between 0.01 and 0.5; however, the particle size increased sharply to ≈376 nm when the N/P ratio reached 2 (Figure 1D). Agarose gel electrophoresis was used to assess the DNA loading capacity of PMet (Figure 1E). Complete DNA encapsulation was achieved at an N/P ratio ≥ 0.1. However, a further increase beyond 0.5 led to a significant particle size enlargement. Thus, an N/P ratio of 0.5 was optimal for preparing PMD nanoparticles, balancing both size and DNA loading efficiency.

Subsequently, Dox was intercalated into dsDNA via hydrogen bonding between the anthraquinone ring of Dox and the DNA bases,^[^ ^19^ ^]^ resulting in dual drug‐loaded nanoparticles, PMDD. Within a Dox concentration range of up to 500 µM, the drug loading efficiency exceeded 80% (Figure 1F). However, increasing the Dox concentration to 800 µM significantly reduced the encapsulation efficiency to 62%, suggesting near‐saturation binding of Dox to DNA at 500 µm. Finally, HA was coated onto the surface of PMDD and PMD to construct PMDDH and PMDH nanoparticles. After HA adsorption, the DLS particle size of PMDDH remained stable (Figure 1G), while the ζ‐potential shifted to a more negative value (Figure 1H). TEM imaging revealed that PMDDH maintained a spherical morphology with a diameter of ≈200 nm (Figure 1I).

UV–vis spectroscopy confirmed the successful binding of Dox and PMet in the nanocarrier, with a characteristic absorption peak of PMDDH near 230 nm corresponding to the guanidine group of PMet and a broad absorption peak specific to Dox between 420 and 560 nm (Figure 1J). FTIR spectroscopy further validated the successful HA modification of PMDDH, with characteristic peaks at 1667.96 cm^−1^ (C═O stretching vibration), 1413.51 cm^−1^ (C─H bending vibration), and 1042.42 cm^−1^ (C─O stretching vibration), consistent with the presence of amide bonds, methyl/methylene groups, and glucose rings (Figure 1K). The drug release behavior of PMDDH was evaluated under different pH conditions. The nanoparticles showed minimal drug release over 48 h at pH 7.4. In contrast, at pH 5.5, the release rate was significantly accelerated, with cumulative release reaching 55% within 24 h (Figure 1L). These results suggest that PMDDH exhibit pH‐responsive drug release, with stable encapsulation under physiological conditions and enhanced release in the acidic intracellular environment, facilitating targeted pharmacological effects.^[^ ^20^ ^]^

### 2.2 Tumor Cell Targeting, Cytotoxicity, and Induction of ICD by PMDDH

**Figure 2:**
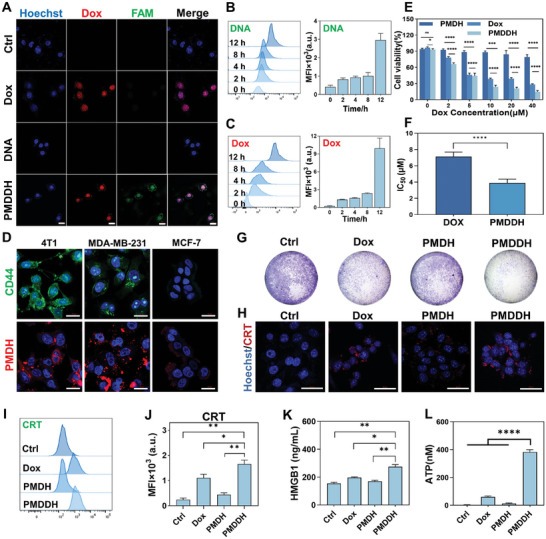
A) Visualization of cellular uptake of Dox, free FAM‐DNA, and PMDDH by CLSM. Scale bar = 20 µm. B,C) Flow cytometry analysis and corresponding quantitative results of PMDDH uptake in 4T1 cells (*n* = 3). D) Visualization of CD44 expression (green) and PMDDH uptake (red) in 4T1, MDA‐MB‐231, and MCF‐7 cells. Scale bar = 20 µm. E) Dose‐dependent cytotoxicity of Dox, PMDH, and PMDDH in 4T1 cells (*n* = 5). F) IC_50_ values of Dox and PMDDH in 4T1 cells (*n* = 5). G) Crystal violet staining of treated 4T1 cells showing cell density and distribution changes. H) CRT expression (red) in 4T1 cells post‐treatment. Scale bar = 50 µm. I) Flow cytometry analysis of CRT expression and J) corresponding quantitative results (*n* = 3). K) HMGB1 levels in the supernatant of 4T1 cells following treatment (*n* = 3). L) ATP release from 4T1 cells after 24 h of different treatments (*n* = 3). Data are presented as mean ± SD (*p < 0.05, **p < 0.01, ***p < 0.001, ****p < 0.0001).

The cellular‐level evaluation of PMDDH was conducted to assess its uptake, targeting ability, cytotoxicity, and capacity to induce ICD in tumor cells. The cellular uptake of PMDDH by 4T1 cells was visualized using confocal laser scanning microscopy (CLSM) (**Figure** **2**A). Free Dox (red fluorescence) rapidly entered the cells and accumulated in the nucleus, consistent with its pharmacological mechanism. In contrast, free DNA labeled with FAM (green fluorescence) displayed limited cellular uptake due to its negative charge. However, PMDDH treatment resulted in intracellular solid green and red fluorescence, demonstrating the nanocarrier's efficient co‐delivery of Dox and DNA. Flow cytometry further confirmed the time‐dependent uptake of PMDDH, which quantified the intracellular fluorescence intensity of Dox (Figure 2B) and DNA (Figure 2C) after various incubation periods with 4T1 cells.

To evaluate the targeting capability of PMDDH post‐HA modification, CD44, a membrane receptor protein, was labeled with FITC (green). We assessed the uptake of PMDDH in 4T1, MDA‐MB‐231, and MCF‐7 cells, which exhibit high, medium, and low levels of CD44 expression, respectively (Figure 2D). Results showed that nanoparticle uptake positively correlated with CD44 expression levels, indicating that PMDDH could specifically target tumor cells through HA‐CD44 recognition.

The anti‐tumor efficacy of PMDDH was primarily evaluated in 4T1 cells by using the CCK‐8 assay (Figure 2E). PMDH demonstrated negligible cytotoxicity across all concentrations, confirming the biocompatibility of the nanocarrier. Compared to free Dox, PMDDH exhibited enhanced cytotoxicity of 4T1 cells with IC_50_ value decreasing by 2‐fold, highlighting the synergistic effect of the PMDH carrier with Dox (Figure 2F). Similarly, PMDDH demonstrated enhanced anti‐tumor activity in both MDA‐MB‐231 and B16‐F10 cell lines, further highlighting its broad applicability in anti‐tumor therapy (Figure S3A,B, Supporting Information). Crystal violet staining was employed to evaluate the changes in cell density and distribution of 4T1 cells following different treatments (Figure 2G). Treatment with Dox and PMDDH resulted in a marked reduction in purple staining, with PMDDH exhibiting the most pronounced effect, thereby demonstrating its potent ability to effectively inhibit tumor cell proliferation.

Previous studies have established that Dox can induce ICD in tumor cells, stimulating anti‐tumor immune responses.^[^ ^21^ ^]^ To evaluate the ICD‐inducing capability of Dox, we measured three key ICD markers: CRT, HMGB1, and ATP.^[^ ^22^ ^]^ CRT exposure on the cell surface acts as an “eat me” signal to immune cells, promoting DCs maturation and enhancing tumor cell phagocytosis. The release of HMGB1 and ATP further boosts tumor cell immunogenicity, recruits immune cells, and promotes their differentiation and antigen presentation. Upon treatment with Dox, 4T1 cells exhibited a significant increase in surface CRT expression (Figure 2H–J), decreased intracellular HMGB1 levels (Figure S4, Supporting Information), and elevated extracellular HMGB1 release (Figure 2K). Additionally, ATP release from cells significantly increased (Figure 2L). PMDDH induced higher expression and release levels of these biomarkers than Dox, indicating its superior ICD‐inducing capability, consistent with the enhanced cytotoxicity observed in the CCK‐8 assays.

### 2.3 In Vitro Anti‐Tumor Immunomodulatory Activity of PMDDH

**Figure 3:**
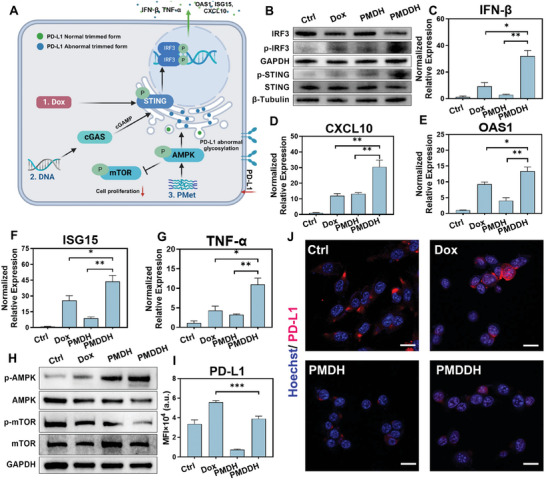
A) Schematic illustration of cGAS‐STING and AMPK signaling activation in 4T1 cells. B) Western blot analysis of p‐IRF3 and p‐STING levels in 4T1 cells after different treatments. RT‐qPCR analysis of cytokine and chemokine expression: C) IFN‐β, D) CXCL10, E) OAS1, F) ISG15, and G) TNF‐α in 4T1 cells. H) Western blot results showing p‐AMPK and p‐mTOR levels in 4T1 cells post‐treatment. I) Flow cytometry analysis and J) immunofluorescence imaging of PD‐L1 expression in 4T1 cells following various treatments. Scale bar = 20 µm. Data are presented as mean ± SD (*n* = 3, *p < 0.05, **p < 0.01, ***p < 0.001).

Beyond direct anti‐tumor effects, the dsDNA and metformin components of PMDDH contribute to immunomodulatory effects, enhancing the overall anti‐tumor response synergistically (**Figure** **3**A). dsDNA activates the cGAS‐STING signaling pathway, which plays a critical role in innate immunity by triggering downstream immune responses.^[^ ^23a,b^ ^]^ Activation of STING leads to the phosphorylation of interferon regulatory factor 3 (IRF3) and IκBα proteins, which subsequently induces the expression of cytokines and chemokines such as interferon‐β (IFN‐β), C‐X‐C motif chemokine 10 (CXCL10), 2'‐5'‐oligoadenylate synthase 1 (OAS1), interferon‐stimulated gene 15 (ISG15), and tumor necrosis factor‐α (TNF‐α).^[^ ^24^ ^]^ These cytokines enhance cellular defenses against pathogens and promote tumor recognition and clearance by the immune system by producing type I interferon.^[^ ^25^ ^]^

Compared to the control group, both Dox and PMDH increased the phosphorylation levels of IRF3 and STING, though to varying extents (Figure 3B). The activation of cGAS‐STING by Dox is primarily linked to its inhibition of topoisomerase II, which induces extensive fragmentation of dsDNA.^[^ ^26^ ^]^ In contrast, PMDH directly delivers dsDNA to achieve similar effects. PMDDH demonstrated the most potent activation of the cGAS‐STING pathway, reflecting the synergistic effect of Dox and DNA co‐delivery. Further evaluation of downstream cGAS‐STING signaling showed elevated expression of key cytokines and chemokines, including IFN‐β (Figure 3C), CXCL10 (Figure 3D), OAS1 (Figure 3E), ISG15 (Figure 3F), and TNF‐α (Figure 3G). The expression patterns of these cytokines were consistent with the phosphorylation levels observed, further confirming the activation of the cGAS‐STING pathway.

Metformin, incorporated into the nanoparticles as part of the polymer PMet, enhances anti‐tumor activity by activating the AMPK signaling pathway. AMPK activation inhibits mammalian target of mTOR function through a cascade reaction, suppressing tumor cell proliferation and growth (Figure 3A).^[^ ^27a,b^ ^]^ Additionally, AMPK activation promotes the degradation of PD‐L1 by inducing abnormal phosphorylation modifications, enhancing the efficacy of tumor immunotherapy (Figure 3A).^[^ ^17^ ^]^ Western blot analysis revealed that PMDH significantly increased AMPK phosphorylation while downregulating mTOR phosphorylation, indicating successful activation of the AMPK‐mTOR signaling axis (Figure 3H). The elevated phosphorylation level of AMPK in the PMDDH group suggests a synergistic activation effect of Dox and PMet within 4T1 tumor cells, highlighting the retained biological activity of metformin in the PMet component.

Following the induction of PD‐L1 expression in 4T1 cells with interferon‐γ (IFN‐γ), PD‐L1 levels were assessed across different treatment groups using Western blot (Figure S5, Supporting Information) and flow cytometry (Figure 3I). Dox further upregulated PD‐L1 expression on the surface of 4T1 cells, consistent with existing literature. However, both PMDH and PMDDH significantly downregulated PD‐L1 expression. Immunofluorescence imaging further corroborated these trends (Figure 3J). These results demonstrate that the individual components of PMDDH contribute to its immunoregulatory effects, including Dox‐induced ICD, dsDNA‐mediated activation of cGAS‐STING, and PD‐L1 downregulation by PMet, collectively exerting a potent anti‐tumor immunomodulatory effect.

### 2.4 Protective Effect of PMDDH Against Dox‐Induced Cardiomyocyte Apoptosis

**Figure 4:**
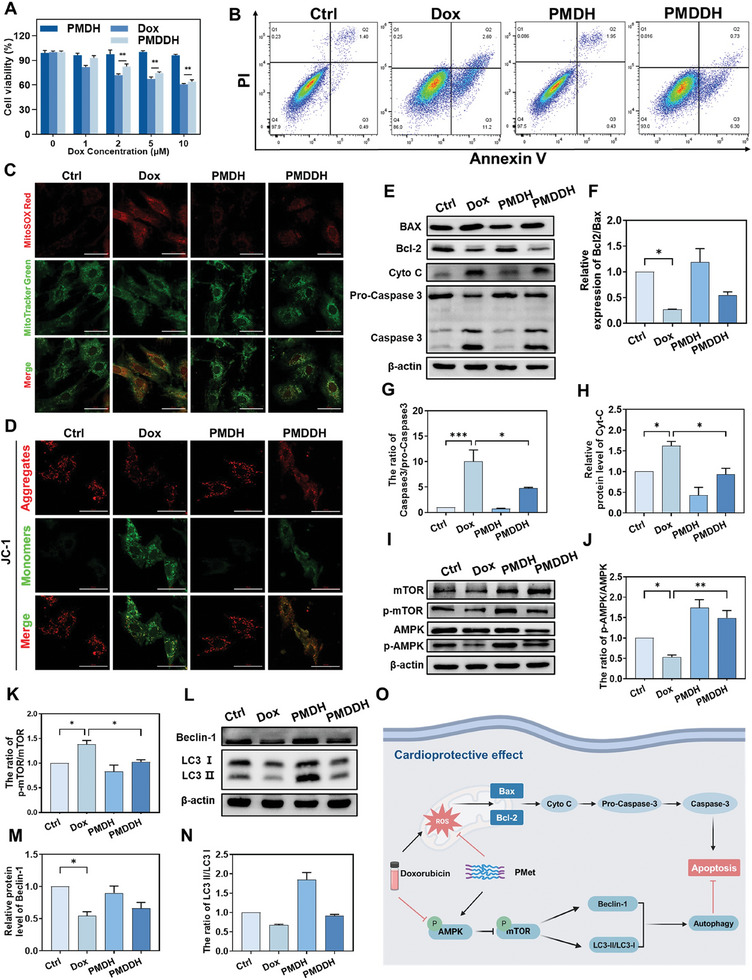
A) Dose‐dependent cytotoxicity of Dox, PMDH, and PMDDH on H9c2 cells (*n* = 5). B) Annexin V‐PI flow cytometry results of apoptosis in treated H9c2 cells. C) MitoSOX Red staining showing mitochondrial ROS levels in H9c2 cells after various treatments. Scale bar = 50 µm. D) JC‐1 staining images depicting mitochondrial membrane potential in H9c2 cells. Scale bar = 50 µm. E) Western blot analysis of apoptosis‐related proteins BAX, Bcl‐2, cytochrome C, and Caspase‐3 in H9c2 cells, and F–H) corresponding quantification (*n* = 3). I) Western blot analysis of AMPK and mTOR, and J,K) corresponding quantification (*n* = 3). L) Western blot of autophagy markers LC3‐I, LC3‐II, and Beclin‐1, and M,N) corresponding quantification (*n* = 3). O) Schematic illustration of the cardioprotective mechanisms of PMDDH in H9c2 cells. Data are presented as mean ± SD (*p < 0.05, **p < 0.01, ***p < 0.001).

The cardioprotective effect of PMDDH against DIC was assessed using H9c2 cardiomyocytes. CCK‐8 assay results demonstrated that PMDH alone did not affect cardiomyocyte viability at various concentrations, indicating its biocompatibility (**Figure** **4**A). In contrast, Dox exhibited dose‐dependent cytotoxicity at concentrations exceeding 1 µm. However, when delivered via PMDDH, the cytotoxicity of Dox toward cardiomyocytes was significantly reduced at the same dose, highlighting the cardioprotective effect of the nanocarrier system. Dox‐induced cardiomyocyte apoptosis, a key factor in cardiac dysfunction, was further evaluated using Annexin V‐PI flow cytometry (Figure 4B). Quantitative analysis showed a significant increase in the apoptosis rate in H9c2 cells treated with free Dox (Figure S6, Supporting Information). In contrast, PMDDH significantly reduced apoptosis, further confirming its ability to mitigate Dox‐induced cardiomyocyte apoptosis.

DIC is primarily associated with mitochondrial dysfunction, characterized by increased ROS production and decreased mitochondrial membrane potential (Δψm), which are pivotal early events in apoptosis.^[^ ^28^ ^]^ MitoSOX Red staining revealed a significant increase in mitochondrial ROS levels following Dox treatment, which was effectively alleviated by PMDDH (Figure 4C). JC‐1 staining was employed to assess Δψm; Dox disrupted mitochondrial function in H9c2 cells, resulting in a decreased Δψm and increased green fluorescence. In contrast, PMDDH treatment preserved mitochondrial integrity, maintaining normal Δψm and protecting against Dox‐induced mitochondrial dysfunction (Figure 4D). These findings indicate that PMDDH exerts anti‐apoptotic effects by preserving mitochondrial function in cardiomyocytes.

Western blot analysis was performed to detect the key apoptosis‐related proteins, including BCL‐2‐associated X protein (BAX), B‐cell lymphoma‐2 (Bcl‐2), and cleaved Caspase‐3 to elucidate further molecular mechanisms underlying PMDDH's anti‐apoptotic effects.  The balance between pro‐apoptotic BAX and anti‐apoptotic Bcl‐2, along with Caspase‐3 activation, plays a critical role in the execution of apoptosis. Elevated ROS levels activate BAX, inhibit Bcl‐2, increase mitochondrial membrane permeability, and facilitate cytochrome C release into the cytoplasm, leading to Caspase‐3 activation. Compared to the control, Dox treatment reduced the Bcl‐2/BAX ratio , promoted cytochrome C release, and upregulated cleaved Caspase‐3 expression (Figure 4E‐H). Conversely, PMDDH attenuated these effects, suggesting that it mitigates mitochondrial damage and downregulates apoptosis‐related proteins by reducing ROS production and preventing Δψm loss, ultimately reversing Dox‐induced cardiomyocyte apoptosis.

As highlighted by the reviewer, previous studies have demonstrated that metformin can induce protective autophagy through the AMPK/mTOR signaling pathway, which helps mitigate DIC.^[^ ^29^ ^]^ For example, Kim et al. showed that high mTOR activity suppresses unc‐51 like autophagy activating kinase 1 (ULK1) activation by phosphorylating Ser757 on ULK1, disrupting the ULK1‐AMPK interaction and inhibiting autophagy.^[^ ^30^ ^]^ Additionally, the lipidation of microtubule‐associated protein 1 light chain 3 (LC3) to form LC3‐II, facilitated by autophagy‐related 3 protein (ATG3) and autophagy‐related 7 protein (ATG7), is a hallmark of autophagosome formation.^[^ ^31^ ^]^ Based on these findings, we hypothesize that PMDDH may initiate protective autophagy by activating the AMPK/mTOR/ULK1 pathway, thereby alleviating DIC. To validate this hypothesis, we examined the activation of key pathway proteins and the expression of autophagy‐related markers. Our Western blot analysis revealed that Dox treatment resulted in a decreased p‐AMPK/AMPK ratio and increased p‐mTOR/mTOR and p‐ULK1/ULK1 ratios, indicating impaired autophagy. However, PMDDH treatment significantly attenuated these Dox‐induced changes (Figure 4I–K; Figure S7A,B, Supporting Information). Moreover, PMDDH treatment reversed the Dox‐induced reduction in the LC3‐II/I ratio and restored the expression of key autophagy‐related proteins, including Beclin1, ATG3, and ATG7 (Figure 4L–N; Figure S7A,C,D, Supporting Information). These results suggest that PMDDH alleviates Dox‐induced mitochondrial damage and cardiotoxicity by activating the AMPK/mTOR/ULK1 signaling axis, thereby promoting protective autophagy (Figure 4O).

### 2.5 In Vivo Pharmacokinetics, Biodistribution, and Anti‐Tumor Activity of PMDDH

**Figure 5:**
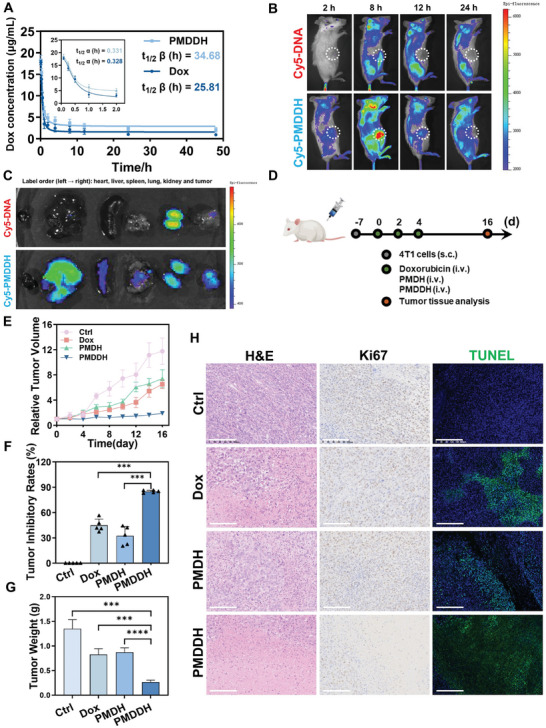
A) Pharmacokinetics of free Dox and PMDDH in rats, with inset showing the drug‐time curve of the distribution phase (*n* = 3). B) In vivo distribution of free Cy5‐DNA compared to Cy5‐labeled PMDDH. C) Ex vivo fluorescence imaging of tumors and major organs in tumor‐bearing mice following intravenous administration of free Cy5‐DNA and PMDDH. D) Schematic of treatment regimen for 4T1 tumor‐bearing mice. E) Tumor growth curves after various treatments over 16 days (*n* = 5). F) Tumor inhibitory rates and G) tumor weights at day 16 (*n* = 5). H) Histological evaluation of tumor tissues using H&E staining, Ki67 immunohistochemistry, and TUNEL immunofluorescence. Scale bars = 100 µm for H&E staining; 200 µm for Ki67 immunohistochemistry and TUNEL immunofluorescence. Data are presented as mean ± SD (*p < 0.05, **p < 0.01, ***p < 0.001).

We then evaluated the in vivo targeting and anti‐tumor efficacy of PMDDH in animal models. Dox and PMDDH were administered to rats via tail vein injection at a dosage equivalent to 5 mg kg^−1^ Dox. Blood samples were collected at designated time points to determine the Dox concentration, and pharmacokinetic parameters were analyzed using a two‐compartment model (**Figure** **5**A). Dox was rapidly distributed with a distribution half‐life (t₁/₂ α) of 0.328 h, attributed to its high lipophilicity. However, the elimination half‐life (t₁/₂ β) of free Dox was ≈25.81 h, whereas PMDDH significantly extended the t₁/₂ β to 34.68 h, an increase of ≈1.3 times. This prolonged circulation time in the PMDDH group demonstrates the nanocarrier's ability to enhance the stability and persistence of Dox efficacy.

To investigate the in vivo biodistribution of PMDDH, Cy5‐labeled DNA was tracked using fluorescence imaging at various time points following intravenous injection (Figure 5B). Free DNA was rapidly cleared and showed minimal accumulation at the tumor site. In contrast, PMDDH exhibited fluorescent solid signals at the tumor site as early as 8 h post‐injection, indicating effective tumor targeting. To further elucidate the distribution of PMDDH in major organs, mice were sacrificed 24 h after treatment, and *ex vivo* imaging was performed on harvested organs and tumor tissues (Figure 5C). The fluorescence intensity in tumor tissue was markedly higher in the PMDDH group compared to free DNA, confirming enhanced tumor‐targeted accumulation. Free DNA predominantly accumulated in the kidneys, suggesting renal metabolism, whereas PMDDH also displayed significant fluorescence in the liver and kidneys, indicating hepatic and renal clearance pathways for the nanoparticles.

The anti‐tumor efficacy of PMDDH was evaluated using 4T1 tumor‐bearing mice randomly assigned to four groups (*n* = 5), treated with PBS, free Dox, PMDH, and PMDDH, respectively (5 mg kg^−1^ Dox dosage; treatment regimen illustrated in Figure 5D). Tumor growth was monitored every two days, and tumor tissues were collected and imaged at the end of the 16‐day treatment period (Figure S8, Supporting Information). Tumor volumes (Figure 5E), tumor weights (Figure 5F), and inhibitory rates (Figure 5G) were measured. Free Dox treatment reduced tumor volumes and significant decreases in tumor mass, demonstrating its anti‐tumor efficacy. PMDH also exhibited some tumor‐suppressive effects, likely due to the immunomodulatory activity of the carrier. Notably, PMDDH achieved the highest anti‐tumor efficacy, with an 85% tumor inhibition rate, confirming the synergistic effect of Dox and the nanocarrier. Histopathologically evaluated tumor tissues using hematoxylin and eosin (H&E) staining, Ki67 immunohistochemistry, and TUNEL immunofluorescence (Figure 5H). PMDDH‐treated tumors showed marked changes in cell density, structural integrity, proliferation, and apoptosis rates, demonstrating its superior therapeutic effects. Overall, these results confirm the enhanced in vivo anti‐tumor activity of PMDDH.

### 2.6 Validation of the In Vivo Anti‐Tumor Immune Enhancement Mechanism of PMDDH

**Figure 6:**
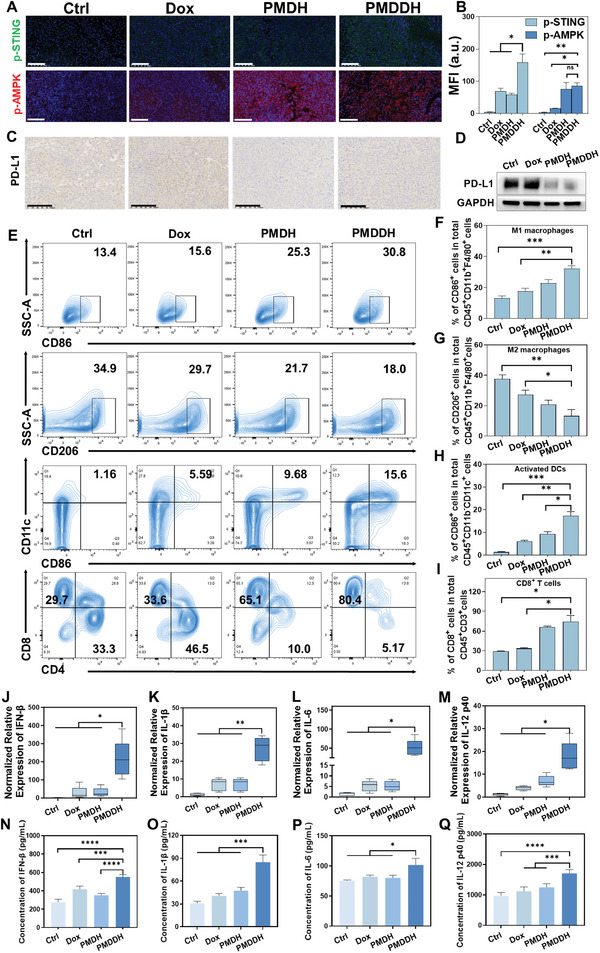
A) Immunofluorescence staining for p‐STING and p‐AMPK in tumors from different treatment groups. Scale bar = 100 µm. B) Quantification of p‐STING and p‐AMPK fluorescence intensity (*n* = 3). C) Immunohistochemical staining of PD‐L1 in tumor tissue. Scale bar = 100 µm. D) Western blot analysis of PD‐L1 expression in tumor tissues from various treatment groups (*n* = 3). E) Flow cytometry analysis of M1 and M2 macrophages, activated dendritic cells (DCs), and cytotoxic T lymphocytes (CTLs) in tumors, with F–I) corresponding quantitative analysis (*n* = 3). J–M) Levels of cytokines IFN‐β, IL‐1β, IL‐6, and IL‐12 p40 in tumor tissues from treated mice (*n* = 5). N–Q) Serum concentrations of IFN‐β, IL‐1β, IL‐6, and IL‐12 p40 following different treatments (*n* = 5). Data are presented as mean ± SD (*p < 0.05, **p < 0.01, ***p < 0.001, ****p < 0.0001).

To investigate the in vivo immune activation effect of PMDDH, we evaluated its ability to modulate the tumor immune microenvironment. Immunofluorescence analysis revealed that all treatment groups activated the AMPK and cGAS‐STING signaling pathways, with PMDDH showing the most pronounced activation (**Figure** **6**A,B). Immunohistochemical (IHC) staining (Figure 6C) and Western blotting (Figure 6D) demonstrated that while Dox upregulated PD‐L1 expression, both PMDH and PMDDH significantly downregulated PD‐L1 levels. Previous in vitro studies confirmed that the nanocarrier could activate the cGAS‐STING pathway via its dsDNA structure and reduce PD‐L1 expression through PMet, achieving a synergistic anti‐tumor immune effect consistent with the in vivo findings.

We further assessed the alterations in the tumor immune microenvironment (Figure 6E). Flow cytometry analysis and quantitative results indicated that PMDDH promoted macrophage polarization toward the anti‐tumor M1 phenotype (Figure 6F,G), activated DCs (Figure 6H), and increased CD8^+^ T cell infiltration (Figure 6I), suggesting that PMDDH enhances adaptive immunity and boosts tumor cell killing. To further validate the immune activation, we quantified immune‐related cytokines, including IFN‐β, interleukin‐1β (IL‐1β), interleukin‐6 (IL‐6), and interleukin‐12 p40 (IL‐12 p40), in tumor tissues and serum using RT‐qPCR (Figure 6J–M) and ELISA (Figure 6N–Q), respectively. PMDDH significantly upregulated these cytokines, underscoring its role in modulating the tumor immune microenvironment. Overall, PMDDH enhances the anti‐tumor immune response by activating the cGAS‐STING and AMPK pathways, effectively transforming “cold tumors” into “hot tumors” more responsive to immunotherapy. This activation enhances antigen presentation and T cell‐mediated immune responses, ultimately amplifying the efficacy of tumor immunotherapy.

### 2.7 Biological Safety Assessment of PMDDH and Its Alleviation of Doxorubicin‐Induced Cardiotoxicity

The biosafety of PMDDH was systematically evaluated to ensure its suitability for therapeutic applications. The body weight of mice was monitored throughout the treatment period as a direct indicator of their general health status (Figure S9, Supporting Information). Mice treated with free Dox exhibited significant weight loss, highlighting the toxic side effects associated with Dox chemotherapy. After the treatment, blood and major organs were collected from the mice for biochemical analysis (Figure S10A, Supporting Information). The free Dox group showed marked increases in key biochemical markers such as alanine aminotransferase (ALT), aspartate aminotransferase (AST), creatinine (CRE), and blood urea nitrogen (BUN), indicating acute hepatorenal toxicity. Histopathological examination through H&E staining revealed toxic pathological changes in major organs, particularly in the heart and liver. In contrast, the PMDDH and blank PMDH carrier groups demonstrated high biosafety, as evidenced by stable body weight, standard blood biochemical indices, and unremarkable histopathological findings, which indicate a significant reduction in the toxic side effects of Dox (Figure S10B, Supporting Information).

**Figure 7:**
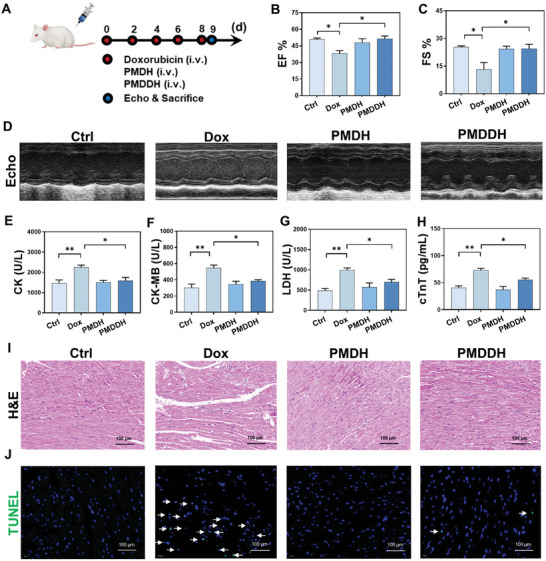
A) Schematic representation of the in vivo cardioprotection experiment. Histograms of B) ejection fraction (EF) and C) fractional shortening (FS) parameters in mice (*n* = 3). D) M‐mode echocardiography images of indicated groups. Serum levels of E) CK, F) CK‐MB, G) LDH, and H) cTnT in treated mice (*n* = 3). I) H&E staining and J) TUNEL staining of myocardial tissue, scale bar = 100 µm. Data are presented as mean ± SD (*p < 0.05, **p < 0.01, ***p < 0.001, ****p < 0.0001).

Among the various adverse effects of Dox, cardiotoxicity is particularly concerning due to its progressive and irreversible nature. Thus, mitigating DIC remains a critical challenge in clinical settings. Our previous in vitro experiments confirmed that PMDDH alleviates DIC by inhibiting cardiomyocyte apoptosis. To validate this effect in vivo, we established a mouse model of DIC (**Figure** **7**A). Echocardiographic assessment showed that free Dox treatment significantly reduced the left ventricular ejection fraction (EF) and fractional shortening (FS), indicating impaired cardiac contractility in mice (Figure 7B–D). Serum biomarkers of myocardial injury, including creatine kinase (CK) (Figure 7E), creatine kinase‐MB (CK‐MB) (Figure 7F), lactate dehydrogenase (LDH) (Figure 7G), and cardiac troponin T (cTnT) (Figure 7H), were all elevated in the Dox group, whereas no abnormalities were observed in the PMDDH group at the same dosage.

Histopathological analysis of cardiac tissue via H&E staining revealed significant structural disarray and inflammatory cell infiltration in the Dox‐treated group, which were notably mitigated by PMDDH (Figure 7I). TUNEL staining further demonstrated that PMDDH significantly reduced the extent of Dox‐induced apoptosis in cardiac tissue (Figure 7J). These findings collectively suggest that PMDDH not only serves as a highly biocompatible delivery system but also effectively reduces the systemic toxicity of Dox, particularly in terms of cardioprotection against Dox‐induced myocardial injury.

## 3 Conclusion

In this study, we successfully designed and evaluated a novel self‐assembled nanomedicine platform, PMDDH, for the co‐delivery of Dox and metformin. The results of our in vitro and in vivo experiments demonstrate that PMDDH not only improves the therapeutic efficacy of Dox but also significantly reduces its cardiotoxicity. Metformin enhances the anti‐tumor effects of Dox by activating the AMPK signaling pathway, downregulating PD‐L1 expression, and promoting ICD. Furthermore, the nanocarrier's design ensures enhanced tumor targeting and immune modulation through the cGAS‐STING pathway, transforming “cold tumors” into “hot tumors” with improved immune responsiveness. Significantly, PMDDH mitigates the cardiotoxicity of Dox by reducing oxidative stress, preserving mitochondrial integrity, and inducing protective autophagy in cardiomyocytes. These findings suggest that PMDDH holds significant potential as a safer and more effective alternative to conventional Dox therapy, offering a promising strategy to enhance the therapeutic index of chemotherapeutic agents.

## 4 Experimental Section

### Materials, Cell Lines and Animals

Synthetic double‐stranded DNA sequences and their complement, along with FAM/Cy5‐labelled counterpart, were procured by Shanghai Bioegene Co., Ltd (Shanghai, China), and the sequences (5′→3′) were: CCAACCAACCAACCAACCAACCAACCAACCAACCAACCAA, and the complementary sequence (5′→3′) was TTGGTTGGTTGGTTGGTTGGTTGGTTGGTTGGTTGGTTGG. The linear polyethylenimine (PEI), dicyandiamide, and Dox were purchased from Sigma‐Aldrich (St Louis, MO, USA) and Zhejiang Hisun Pharmaceutical Co., Ltd. (Taizhou, China). Hyaluronic acid was obtained from Aladdin Scientific Corp (Shanghai, China). The Dulbecco's Modified Eagle Medium (DMEM), Dulbecco's Modified Eagle Medium/Nutrient Mixture F‐12 (DMEM/F‐12), and Roswell Park Memorial Institute (RPMI)‐1640 medium were acquired from Gibco Life Technologies, Inc. (Grand Island, NY, USA). The fetal bovine serum was purchased from ExCell Bio Group (Shanghai, China). The penicillin‐streptomycin liquid, trypsin, Hoechst 33342 stain, 4′, 6‐diamidino‐2‐phenylindole (DAPI), 10% normal goat serum, and protein phosphatase inhibitor were supplied by Beijing Solarbio Science & Technology Co., Ltd (Beijing, China). The PBS, CCK‐8 assay kit, crystal violet staining solution (0.1%), RIPA lysis buffer, phenylmethanesulfonyl fluorideand (PMSF), and 5×SDS‐PAGE loading buffer were purchased from Labgic Technology Co., Ltd (Anhui, China). The RevertAid First Strand cDNA Synthesis Kit was sourced from Thermo Fisher (MA, USA). GelStain and TransStart Green qPCR SuperMix were obtained from TransGen Biotech Co., Ltd (Beijing, China). Bovine serum albumin (BSA) and agarose were purchased from BioFroxx (Einhausen, Germany). IFN‐γ was bought from bio‐techne (Minneapolis, MN, USA). The enhanced ATP assay kit, Mito‐Tracker Green, and enhanced mitochondrial membrane potential assay kit with JC‐1 were supplied by Beyotime Technology (Shanghai, China). MitoSOX Red Mitochondrial Superoxide Indicator was purchased from Shanghai Maokang Biotechnology Co., Ltd. (Shanghai, China), and Tumor Tissue Digestion Solution was obtained from bioGenous (Suzhou, China). FITC Annexin V Apoptosis Detection Kit was purchased from BD Biosciences (New York, USA). ELISA assay kits for cytokines (IL‐6, IFN‐β, IL‐1β, IL‐12 p40, and HMGB1) were obtained from Meimian Industrial Co., Ltd. (Jiangsu, China) or Shanghai Jianglai Industrial (Shanghai, China). The PE‐conjugated‐anti‐PD‐L1 antibody was purchased from Elabscience Biotechnology Co., Ltd (Wuhan, China). Primary antibodies targeting phospho‐STING (Ser365, Cat. #72971), phospho‐IRF3 (Ser396, Cat. #29047), phospho‐AMPKα (Thr172, Cat. #2535), phospho‐mTOR (Ser2448, Cat. #5536), LC3B, Beclin‐1, Cyto C and ATG3 were purchased from Cell Signaling Technology (Beverly, Massachusetts). Primary antibodies for STING, IRF3, AMPK, Calreticulin (CRT), and β‐Tubulin were obtained from Proteintech Group (IL, USA). Anti‐GAPDH was obtained from Huabio (Zhejiang, China). Anti‐mTOR and anti‐ULK were bought from Abmart Shanghai Co., Ltd. (Shanghai, China), and the anti‐CRT‐FITC was bought from Bioss Antibodies (MA, USA). The antibodies targeting phospho‐ULK (Ser757, Cat. AF4387) and ATG7 were obtained from Affinity Biosciences (Jiangsu, China) and Selleck Chemicals LLC (TX, USA), respectively. Antibodies for Bcl‐2, Bax, Caspase‐3 and Ki67 were from Abcam (Cambridge, UK), while secondary antibodies (conjugated with HRP, FITC, or Alexa Fluor 594) were obtained from Zen‐Bioscience Co., Ltd (Sichuan, China). All the primers (HPLC purification) of IFN‐β, OAS1, ISG15, IL‐6, CXCL10, TNF‐α, IL‐1β, and GAPDH were synthesized by Sangon Biotech (Shanghai, China) or Shanghai Bioegene Co., Ltd (Shanghai, China), and the primer sequences were listed in **Table** **1**.

**Table 1:** The primer sequences used in RT‐qPCR.

Gene	Forward primer (5′→3′)	Reverse primer (5′→3′)
IFN‐β	CTGGGTGGAATGAGACTATTGT	AAGTTCCTGAAGATCTCTGCTC
OAS1	TGCATCAGGAGGTGGAGTTTG	ATAGATTCTGGGATCAGGCTTGC
ISG15	GTGCTCCAGGACGGTCTTAC	CTCGCTGCAGTTCTGTACCA
IL‐6	CAAAGCCAGAGTCCTTCAGAG	GTCCTTAGCCACTCCTTCTG
CXCL10	CAACTGCATCCATATCGATGAC	GATTCCGGATTCAGACATCTCT
TNF‐α	ATGTCTCAGCCTCTTCTCATTC	GCTTGTCACTCGAATTTTGAGA
IL‐1β	ATGAAGGGCTGCTTCCAAAC	TCTCCACAGCCACAATGAGT
IL‐12 p40	GAGCACTCCCCATTCCTACT	GCATTGGACTTCGGTAGATG
GAPDH	GGGTCCCAGCTTAGGTTCAT	CCAATACGGCCAAATCCGTT

4T1, H9c2, MCF‐7, MDA‐MB‐231, and B16‐F10 cell lines were provided from Xiangya Cells Center (Changsha, China), cultured, and grown in RPMI‐1640/DMEM F‐12/DMEM medium (GIBCO, USA). All the mediums were supplied with 10% FBS, and 1% streptomycin/penicillin (100 U/mL), and cells were incubated in a humidified atmosphere with 5% CO_2_ at 37 °C.

The BALB/c mice and Sprague‐Dawley rats were purchased from Hunan SJA Laboratory Animal CO., LTD (Hunan, China), housed under specific pathogen‐free conditions. All the animal experiments were performed following the Regulations for the Administration of Affairs Concerning Experimental Animals of China and has been approved by the Institutional Animal Care and Use Committee (IACUC) of Central South University (No. CSU‐2022‐0583 and No. CSU‐2022‐0609).

### Synthesis and Characterization of PMet and PMD

To synthesize the PMet, linear PEI hydrochloride (0.2 g) and dicyandiamide (2 g) were dissolved in 10 mL 2 M hydrochloric acid (HCl) solution. The mixture was then heated at 100 °C for 36 h. Post‐reaction, the raw product was collected and purified by dialyzing (3500 Da) against water for 72 h with frequent replacement of dialysis media to remove unreacted dicyandiamide. Finally, the obtained solution was lyophilized for 24 h. ^1^H NMR (AVANCE III 400 M, Bruker, USA) and MALDI‐TOF mass spectrometry (Ultraflextreme, Bruker, USA) analysis verified the chemical structure of the final product.

For the preparation of PMD, a set amount of PMet stock was added to an aqueous solution containing a fixed concentration of nucleic acid for 30‐minute incubation at room temperature (RT), characterizing the size distribution respectively to determine the desired N/P charge ratio. The PMD was examined by 2% agarose gel electrophoresis to determine the nucleic acid loading efficiency.

### Doxorubicin Loading of PMDDH

To optimize the loading efficiency of Dox into PMDDH, varying concentrations of Dox were pre‐mixed with an equivalent amount of DNA. The mixture was incubated at RT to allow for the formation of Dox‐DNA complexes. Initial trials were conducted at Dox concentrations ranging from 50 to 800 µm. The loading efficiency was evaluated by quantifying the percentage of Dox encapsulated within the nanoparticles.

### Preparation and Characterization of PMDDH

Briefly, 100 µM DNA (5 µL) and 5 mm Dox solution (50 µL) were mixed for 24‐hour incubation at RT, then an additional 43 µL of 1.2 mg mL^−1^ PMet solution and 402 µL ddH_2_O were added, followed by incubation at RT for 15 min. Finally, the mixture was incubated with 500 µL of 4.8 mg mL^−1^ HA solution for 30 min. The ZetaSizer Nano ZS (Malvern Instruments, UK) was used to measure the particle size and ζ‐potential. The morphology of PMDDH was observed by TEM (Titan G2‐F20, FEI, USA). The UV−vis absorption spectra of PMet, HA, Dox, PMDD, and PMDDH were measured by a UV−vis spectrophotometer (U‐4100, Hitachi, Japan). Fourier‐transform infrared spectroscopy (FTIR, Spectrum Two, PerkinElmer) was used to analyze Dox, PMet, HA, PMDH, and PMDDH powder form.

### In Vitro Dox Release Behavior

The release profile of Dox from PMDDH was evaluated using the centrifugation method. Briefly, PMDDH were added to 10 mm phosphate buffer (pH 5.5 and 7.4). The solution was shaken in a thermostat water bath (100 rpm, 37 °C). The release medium was sampled at a predetermined time point and centrifuged at 16 000 rpm for 10 min. The fluorescence intensity (Ex: 485 nm, EM: 600 nm) of the supernatant was measured by a microplate reader (Infinite M200 PRO, TECAN, Austria) to quantify Dox.

### Cellular Uptake Study

The 4T1, MDA‐MB‐231, and MCF‐7 cells were seeded in a 35 mm culture dish. After being treated with FAM‐labeled or Cy5‐labeled PMDDH for 8 h, the cells were fixed with 4% paraformaldehyde, stained with FITC‐cojugated‐anti‐CD44 and Hoechst 33342 to visualize the expression of CD44 and nuclei. Fluorescence distribution was imaged by a confocal laser scanning microscopy (LSM780 NLO, Zeiss, Oberkochen, Germany). Flow cytometry was performed to quantify the PMDDH internalization (FACSVerse, BD, USA).

### In Vitro Cytotoxicity Study

The CCK‐8 assay was conducted to assess the in vitro cytotoxicity. 4T1, MCF‐7, H9c2, MDA‐MB‐231, and B16‐F10 cells were seeded into 96‐well plates and incubated overnight. The cells were then treated with different formulations at varying concentrations for 24 h. Following the treatment, cell viability was measured using the CCK‐8 assay, and the absorbance was recorded with a microplate reader (Infinite M200 PRO, TECAN, Austria). In parallel, treated 4T1 cells were subjected to crystal violet staining to assess cell invasion, and the images were captured under a microscope (TieS, NIKON, Tokyo, Japan). Additionally, the H9c2 cells were seeded into 6‐well plates, and FITC Annexin V Apoptosis Detection Kit was employed to evaluate the apoptosis rates after exposure to the different treatments.

### **Induction of ICD** Study

Immunofluorescence was performed to observe the distribution of CRT on cellular membranes. 4T1 cells were seeded in a 35 mm culture dish and treated with various formulations for 24 h. Cells were stained with anti‐CRT antibody, followed by staining with Alexa Fluor 594 conjugated goat‐anti‐rabbit secondary antibody and 10 µg mL^−1^ Hoechst 33342 for 30 min to visualize the nucleus and CRT. Finally, cells were observed under a confocal laser scanning microscopy (LSM780 NLO, Zeiss, Oberkochen, Germany). To further quantitatively analyze the expression of CRT, FITC‐conjugated anti‐CRT antibodies were added to cell suspension. After that, cells were analyzed by flow cytometry (LSRFortessa, BD, USA). Meanwhile, the content of HMGB1 and ATP in cellular supernatant were measured by test kits.

### Western Blot

The exacted total proteins (20 µg) were separated on SDS‐PAGE gels and transferred to PVDF membranes. Afterward, the membranes were blocked for 1 h and probed with each respective primary antibody at 4 °C overnight, followed by incubation with secondary antibodies at room temperature for 1 h. Protein bands were visualized by ChemiDoc XRS + system (Bio‐Rad, USA).

### Quantitative Real‐Time PCR (RT ‐qPCR)

Total RNA was isolated from cells and tumor homogenates using TRIzol (Biotech, USA). To build a cDNA library, a SuperScript IV First‐Strand Synthesis System reverse transcribed total RNA (500 ng). Real‐time PCR was conducted with the TransStart Green qPCR SuperMix using a Bio‐Rad CFX Connect Real‐time System (Bio‐Rad, USA). Relative RNA abundances were calculated by the standard 2^−ΔΔCt^ method.

### PD‐L1 Degradation

IFN‐γ was added to 4T1 cells for 24 h to induce high expression of PD‐L1 on the cellular membrane. After that, cells were treated with Dox, PMDH, and PMDDH (equivalent Dox dose of 10 µm) for another 24 h, stained with anti‐PD‐L1 antibody and Alexa Fluor 594 conjugated goat‐anti‐rabbit secondary antibody, and observed under a confocal laser scanning microscopy (LSM780 NLO, Zeiss, Oberkochen, Germany). Meanwhile, the 4T1 cells were collected and resuspended in a Cell Staining Buffer (Elabscience, Wuhan, China). PE‐conjugated‐anti‐PD‐L1 antibody was added for 30 min at 4 °C. After that, cells were analyzed by flow cytometry (LSRFortessa, BD, USA) to assess the expression of PD‐L1. Western Blot assay also measured the included PD‐L1 from total cellular protein.

### Mitochondrial Membrane Potential (MMP) and ROS Production

The mitochondrial membrane potential (MMP) of H9c2 cells treated with Dox, PMDH, or PMDDH (equivalent Dox dose of 10 µM) was assessed using the Enhanced Mitochondrial Membrane Potential Assay Kit with JC‐1. Mitochondrial ROS production in treated H9c2 cells was visualized using Mito‐Tracker Green and MitoSOX Red Mitochondrial Superoxide Indicator. Fluorescence signals were detected using a confocal laser scanning microscopy (LSM780 NLO, Zeiss, Oberkochen, Germany).

### Pharmacokinetics Study

The mice were injected with free Dox or PMDDH intravenously at a Dox equivalent dose of 5 mg kg^−1^. The orbital blood samples were collected at predetermined time points and centrifuged at 3000 rpm for 10 min to separate serum. The content of Dox in serum was quantified by fluorescence (Ex: 485 nm; Em: 600 nm). The blood circulation followed a typical two‐compartment model: a rapid decline in the distribution and longer elimination phases.

### Biological Distribution

The mice were intravenously injected with Cy5‐labeled free DNA or PMDDH (equivalent DNA dose of 1.3 mg kg^−1^). The mice were imaged using the IVIS Lumina III Imaging System at the indicated time points after injection. After 24 h, the mice were sacrificed, and the tumors and organs (including heart, liver, spleen, lung, and kidney) were harvested for ex vivo imaging (PerkinElmer, Shanghai, China) (Ex: 535 nm; Em: 610 nm).

### In Vivo Anti‐Tumor Activity

The 4T1 tumor‐bearing BALB/c mice were randomly assigned into four groups (*n* = 5 per group) as follows: (1) Ctrl; (2) Dox; (3) PMDH; (4) PMDDH; The Dox, PMDH, and PMDDH (Dox 5 mg kg^−1^) were administrated intravenously every 2 days for overall three times. The tumor sizes and body weight were recorded every two days. The tumor volume was calculated by the following formula: V (mm^3^) = (width^2^ × length) /2. Tumors and major organs (heart, liver, spleen, lung, and kidneys) were harvested and immersed in 4% formaldehyde, followed by embedding and slicing. Finally, the slides were stained with H&E, Ki67, TUNEL, anti‐p‐STING antibody, anti‐p‐AMPK antibody and anti‐PD‐L1 antibody for imaging, respectively. The levels of cytokines including IFN‐β, IL‐1β, IL‐6 and IL‐12 p40 in mice's serum was determined by ELISA kits according to the instructions.

### Tumor‐Infiltrating Lymphocytes Analysis

The harvested tumors were cut into small pellets, digested in a thermostatic shaker (50 rpm, 37 °C) for 1 h using Tumor Tissue Digestion Solution and filtered through 70 µm cell strainers (Falcon, NY, USA) to obtain single‐cell suspensions. After the erythrocyte lysis procedure by 1×ACK Lysis Buffer (Elabscience, Wuhan, China), added anti‐mouse CD16/32 antibody to block FcγIII/IIR, and then cells were stained with fluorescence‐conjugated antibodies by turn. The following antibodies used for flow cytometry were purchased from Elabscience Biotechnology Co., Ltd (Wuhan, China). The anti‐CD45‐Alexa Fluor700 or Elab Fluor Violet 450 anti‐mouse CD45 antibody was used to sort total immune cells. For macrophage repolarization, the cells were stained with Elab Fluor Red 780 anti‐mouse/human CD11b antibody, Elab Fluor Violet 450 anti‐mouse F4/80 antibody, FITC anti‐mouse CD86 antibody and APC anti‐mouse CD206/MMR antibody. For DCs maturation, the cells were stained with Elab Fluor Red 780 anti‐mouse/human CD11b antibody, APC anti‐mouse CD11c antibody, and FITC anti‐mouse CD86 antibody. For T cell activation, the cells were stained with Elab Fluor Red 780 anti‐mouse CD3 antibody, FITC anti‐mouse CD4 antibody, and APC anti‐mouse CD8a antibody. After incubating at 4 °C, the samples were analyzed by flow cytometry (LSRFortessa, BD, USA).

### In Vivo Cardioprotective Effect

Forty BALB/c mice (8 weeks, 20–25 g) were randomly and equally divided into 4 groups (*n* = 10): (1) Ctrl, (2) Dox, (3) PMDH, and (4) PMDDH. Mice in the Dox group were intravenously administered 3 mg kg^−1^ Dox every other day 5 times (total dose: 15 mg kg^−1^). Mice in the PMDH and PMDDH were injected with corresponding NPs equivalent to Dox by tail vein. One day after the last injection, all mice were deeply anesthetized by isoflurane and fixed on a platform, with chest hairs removed. Echocardiography was performed using a Vevo2100 ultrasound real‐time imaging system (FUJIFLM Visualsonis, Canada) to obtain functional parameters in a blinded manner. Then, the heart samples were fixed and sliced, and then stained with H&E and TUNEL for detection.

### Blood Biochemical Index Analysis

After the in vivo anti‐tumor activity experiment, the blood samples of the mice were collected from eyeballs, and serums were obtained through centrifugation at 3000 rpm for 10 min for biochemical analyses. The toxicity of the liver (ALT, AST), kidney (BUN, CRE), and heart (CK, CK‐MB, LDH, cTnT) were measured by a 7600 automated biochemistry analyzer (HITACHI, Tokyo, Japan).

### Statistical Analysis

All the quantitative results were reported as mean ± standard deviation (SD). The one‐way analysis of variance (ANOVA) and Student t‐test were used to assess the differences among multiple groups. The significance threshold was defined as: *p < 0.05, **p < 0.01, ***p < 0.001, ****p < 0.0001.

## Conflict of Interest

The authors declare no conflict of interest.

## Supporting information



Supporting Information

## Data Availability

The data that support the findings of this study are available from the corresponding author upon reasonable request.
