# Supplementary material for: Mitigating Doxorubicin‐Induced Cardiotoxicity and Enhancing Anti‐Tumor Efficacy with a Metformin‐Integrated Self‐Assembled Nanomedicine
Source: Adv Sci (Weinh). 2025 Mar 7;12(17):2415227. doi: 10.1002/advs.202415227 (PMC12061326; doi:10.1002/advs.202415227)
Supplement: Supplementary file 1 — Supporting Information [file ADVS-12-2415227-s001.docx]

Supporting information for

**Mitigating Doxorubicin-Induced Cardiotoxicity and Enhancing Anti-Tumor Efficacy with a Metformin-Integrated Self-Assembled Nanomedicine**

*Jiaxin Huang^1, 2#^, Jieru Yang^2#^, Yuanying Yang^1#^, Xiaofeng Lu^3, 4^, Juan Xu^2, 3^, Shan Lu^2^, Hong Pan^2^, Wenhu Zhou^2, 5^*, Wenqun Li^1^* and Songwen Chen^3^**

^1^ Department of Pharmacy, Second Xiangya Hospital, Central South University, Changsha, Hunan, 410011, China

^2^ Xiangya School of Pharmaceutical Sciences, Central South University, Changsha, Hunan, 410013, China.

^3^ Department of Cardiology, Shanghai General Hospital, Shanghai Jiao Tong University School of Medicine, No.100, Haining Rd, Shanghai, 200080, China.

^4^ Department of Cardiology, Shanghai General Hospital Jiuquan Hospital, No. 22, West St, Jiuquan, Gansu, 735000, China.

^5^ Hunan Key Laboratory of The Research and Development of Novel Pharmaceutical Preparations, School of Pharmaceutical Science, Changsha Medical University, Changsha, Hunan, 410219, China

Corresponding author. Email: zhouwenhu@csu.edu.cn; liwq1204@csu.edu.cn; chensongwen@shsmu.edu.cn

**Figure S1.** (A-B) ^1^H NMR spectra and (C) MALDI-TOF mass spectra of PEI and PMet.

**Figure S2.** Gel permeation chromatography (GPC) characterization of the mean molecular weight of PMet.

**Figure S3.** The dose-dependent cytotoxicity of (A) MDA-MB-231 and (B) B16-F10 cells (n = 5).

**Figure S4.** The expression of HMGB1 in 4T1 cells after various treatments.

**Figure S5.** The expression of PD-L1 in 4T1 cells after various treatments.

**Figure S6.** The apoptosis rate of treated H9c2 cells (n = 3).

**Figure S7.** (A) Western blot of autophagy markers p-ULK/ULK, ATG7, and ATG3, and (B-D) corresponding quantification (n = 3).

**Figure S8.** The tumor tissues harvested on Day 16 after different treatments.


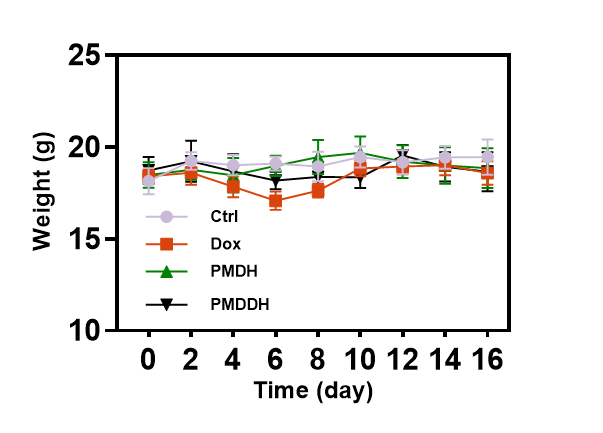


**Figure S9.** Average body weights of mice with different treatments (n = 5).

**Figure S10.** (A) The level of biochemical indexes, including ALT, AST, Cre, and BUN, in the mice's serum (n = 5). (B) Histology images of H&E staining slices for major organs harvested from mice with different treatments. Scale bar = 100 μm
